# Concentrations, Speciation, and Potential Release of Hazardous Heavy Metals from the Solid Combustion Residues of Coal-Fired Power Plants

**DOI:** 10.3390/ijerph191912617

**Published:** 2022-10-02

**Authors:** Yiming Huang, Jinling Liu, Guan Wang, Xiangyang Bi, Guangyi Sun, Xian Wu, Qingfeng Wang, Zhonggen Li

**Affiliations:** 1 School of Resources and Environment, Zunyi Normal College, Zunyi 563006, China; 2 School of Earth Sciences, China University of Geosciences, Wuhan 430074, China; 3 MOE Laboratory for Earth Surface Processes, College of Urban and Environmental Sciences, Peking University, Beijing 100871, China; 4 State Key Laboratory of Environmental Geochemistry, Institute of Geochemistry, Chinese Academy of Sciences, Guiyang 550081, China

**Keywords:** heavy metal, coal fly ash, desulfurization gypsum, leachability, sequential extraction, pollution

## Abstract

Hazardous heavy metal-laden coal combustion byproducts exposed to precipitation or surface/groundwater are of environmental concern. This study analyzed fly ash (FA) and desulfurization gypsum (FGD gypsum) samples obtained from 16 coal-fired power plants in Guizhou Province, China. A combination of field and laboratory studies was used to investigate the binding forms of lead (Pb), cadmium (Cd), and chromium (Cr) and their leaching characteristics under natural storage conditions. The results showed that there were significant proportions of residual states of these elements in FA (84–99% for Pb, 83–91% for Cd, and 73–97% for Cr), indicating a lack of migration to other environmental media. FGD gypsum contained high proportions of metals in migratable states, but the environmental risks were low due to their very low concentrations. The release of Pb, Cd, and Cr from FA and FGD gypsum into extracts varied according to pH. This is related to the form of each element in the solid and the series of reactions that occurs during the leaching process. However, aside from Cr in FA, all heavy metals in FA and FGD gypsum samples were present in concentrations below the relevant standards for landfill leachate, indicating very low release rates. The Cr levels (206–273 μg/L) in some of the FA extracts were higher than the limits for water pollution from domestic landfill, indicating that Cr in FA poses a leaching risk. The results of field leachate sampling and indoor simulated rainfall experiments further validated these findings, indicating that the release of Cr from stockpiled coal FA is a cause for concern.

## 1. Introduction

Heavy metals pose great threats to the natural environment and human health due to their biodegradability, biotoxicity, and ability to bioaccumulate. Hazardous heavy-metal pollution is dangerous and irreversible and has long been a hot topic in environmental research. In recent years, human industrial production has resulted in increased emissions of heavy metals from anthropogenic sources, leading to significant increases in heavy-metal concentrations on the Earth’s surface and in the atmosphere [1,2,3]. Coal combustion is one of the most important sources of heavy-metal emissions globally [4,5] and produces both direct atmospheric emissions and secondary cross-media transport. Therefore, controlling the dispersion of heavy metals from coal-fired power plants (CFPPs) is essential in reducing heavy-metal contamination. According to previous studies, atmospheric emissions of heavy metals from CFPPs are now effectively controlled [6,7]. Almost all heavy metals produced by CFPPs are captured as solid byproducts such as coal fly ash (FA) and desulfurization gypsum (FGD gypsum). Bottom ash is another major coal combustion byproduct, but it is mainly in a molten state and there is a very low environmental risk of leaching [8]; therefore, it was not considered in this paper.

The current treatment methods for coal combustion byproducts include stockpiling and reutilization. During natural stockpiling, solar irradiation can cause small amounts of volatile elements to escape into the atmosphere, while rainfall can also leach heavy metals into the soil and groundwater. The overall secondary utilization rate of FA and FGD gypsum in China is currently around 70% [9] and is even lower in certain underdeveloped regions such as Guizhou Province. Therefore, large amounts of coal combustion byproducts have become concentrated in landfills; these contain high concentrations of heavy metals that are gradually released into the environment. In terms of reutilization, FA is often mixed with agricultural soil to loosen the soil and increase its air tightness, or it is applied as a fertilizer [10,11]. During this utilization process, hazardous heavy metals in solid combustion residues can be released directly into the environment and are absorbed by agricultural products, thus posing significant risks to humans.

Previous studies have examined the cross-media migration potential of heavy metals in solid byproducts of CFPPs, with most studies focusing on mercury (Hg) [12,13,14,15,16,17]. Świetlik et al. investigated the fate of six hazardous metals, including cadmium (Cd), copper (Cu), lead (Pb), and zinc (Zn), in FA from three CFPPs with electrostatic precipitators located in Poland [18]. The heavy metals in FA were mainly present in environmentally persistent (i.e., residual) states (40–90%). In a study by Quispe et al. on the forms and release properties of heavy metals in FA from the Santa Catarina power plant in Brazil, the proportions of mobile heavy-metal forms in successive extraction data, which were weighted by the annual production, were used to estimate the total amounts of pollutants emitted from the plant per year. The results indicated that approximately 9.1 tons of Cr and 1.5 tons of Pb were emitted and could affect the surrounding environment [19]. In addition, 30–40% of the FA was found to be soluble in Cr, compared to 25% in Cu and 1% in Pb. Zhao et al. investigated the distribution patterns and leaching characteristics of seven hazardous heavy metals, including Hg, Cr, Cd, and Pb, in FA and FGD gypsum from four powerplants in China [12,20]. The leaching concentration of Cr in some FA samples under strongly alkaline conditions was above the permissible limit. Although the leaching experiments carried out in this study indicated that the FGD gypsum samples could be considered nonhazardous waste, it was suggested that reasonable disposal measures are still needed to reduce the risk of releasing hazardous heavy metals given the large amounts of coal combustion byproducts being produced and ending up in landfill.

Most of the above studies were laboratory studies, with very few field studies in the published literature. Studies of hazardous heavy-metal elements such as Pb, Cd, and Cr in FA and FGD gypsum are relatively sparse, fragmented, and unsystematic. Guizhou is the fourth-largest coal-producing province in China [21], and its CFPPs output 35 million tons of FA and FGD gypsum annually. Over 150 million tons have been deposited over the years, covering an area of over 130 ha [22,23]. While the average national secondary utilization rate of FA and FGD gypsum is about 70% [9], the comprehensive utilization rate in Guizhou Province is significantly lower at 38% [22], and its coal combustion by-products cause greater environmental risk as they are stockpiled in the natural environment. In addition, Guizhou Province is located in the center of China’s karst landscape, which covers 62% of the province’s land area [24], and the subtropical monsoon climate of the plateau means that there is abundant rainfall (1191 mm/year) in the region. In recent years, the rainfall has been acidic [25], and the low rate of soil formation and high permeability of carbonate rocks have led to rock desertification and soil erosion, making the ecological environment very fragile [26]. There has been inadequate research on this topic in Guizhou Province and, as such, there is an urgent need for environmental fate studies on heavy metals in the solid byproducts resulting from flue gas treatment in Guizhou’s CFPPs.

On the basis of previous work [12,13,14,15,16,17], a systematic study combining field observations and indoor simulation studies was performed on FA and FGD gypsum samples generated from coal of various qualities burnt in different furnace types with various combinations of pollution control facilities in Guizhou Province. Since the differences in the volatility of heavy metals underlie their significant behavioral differences in CFPPs, three heavy metals (Pb, Cd, and Cr) were examined according to their toxicity. The aims of the present study were to (1) explore the binding forms of Pb, Cd, and Cr in FA and FGD gypsum from CFPPs, (2) determine the leaching characteristics of Pb, Cd, and Cr in FA and FGD gypsum under different situations, such as varying pH conditions and solid–liquid ratios, and (3) identify the environmental release of Pb, Cd, and Cr from FA and FGD gypsum samples under stockpile conditions in the field. The results of this study provide a systematic and comprehensive picture of the environmental threats posed by the heavy metals found in the solid byproducts of CFPPs. Furthermore, this study provides a theoretical basis and empirical support for the control of heavy-metal emissions from CFPPs.

## 2. Materials and Methods

### 2.1. Sequential Extraction of Various Fractions of Heavy Metals

#### 2.1.1. CFPPs and Sample Collection

An experimental study was conducted on the species of Pb, Cd, and Cr in FA and FGD gypsum samples from 16 CFPPs in Guizhou Province. All CFPPs were fed with local coal, which has double the ash yield and sulfur concentration with respect to the national average [7,27]. Moreover, the ash yield and sulfur concentration are higher in eastern Guizhou than western Guizhou. All solid samples were collected on 3–6 occasions from each plant, with a half-day sampling interval and a minimum sample size of 1 kg. Most CFPPs were distributed in the central to western areas of Guizhou Province, which are the main coal production areas. Detailed information about the CFPPs is shown in Table 1.

#### 2.1.2. Determination of Pb, Cd, and Cr in Solid Samples

FGD gypsum samples were oven-dried at 40 °C and ground to particles of <0.15 mm in size; FA samples were dry when sampled and were not ground. The sample digestion method developed by [28] was used. Briefly, 50 mg powder samples were weighed in Teflon digestion bottles, 1 mL of hydrofluoric acid (HF) and 1 mL of nitric acid (HNO_3_) were added, and then the samples were placed in an oven preheated to 190 °C for 24 h. After cooling, each sample was heated on a hotplate at 120 °C to evaporate the solution to incipient dryness. Subsequently, 0.5 mL of HNO_3_ was added to each Teflon bottle, and the samples were continuously heated on a hotplate until dry. Then, 200 ng of Rh (as an internal standard), 2 mL of HNO_3_, and 2 mL of deionized water were added sequentially, and the samples were placed in an oven preheated to 150 °C for 5 h. Finally, 0.4 mL of each digestion solution was transferred into a 15 mL centrifuge tube, and deionized water was added to obtain a volume of 10 mL. The Pb, Cd, and Cr in each solution was measured using inductively coupled plasma mass spectrometry (ICP-MS; Analytik Jena, Germany). The data for the solid samples are reported on the basis of their air-dried masses.

#### 2.1.3. Sequential Extraction Experiment 

The stepwise sequential extraction method proposed by the Community Bureau of Reference (BCR) was used to extract Pb, Cd, and Cr in various forms from the FA and FGD gypsum [29]. Detailed information on this process is shown in Table 2, and a description of the sequential extraction method is presented in the Supplementary Materials. 

### 2.2. Heavy-Metal Leaching Experiment

FA samples from CFPP #1 (P1E1, P1E2), CFPP #2 (P2F1, P2F2), and CFPP #3 (P3E1, P3E2; Table 1) and FGD gypsum samples from CFPP #7 (P7D1, P7D2), CFPP #11 (P11D1, P11D2), CFPP #12 (P12D1, P12D2), and CFPP #16 (P16D1, P16D2, P16D3, P16D4) were selected for investigation of the leaching of heavy metals from FA and FGD gypsum. These selections were based on the regions of the CFPPs, the types of boilers used, the pollutant control facilities, the sampling locations, and the solid byproduct heavy-metal contents.

**Figure 1:**
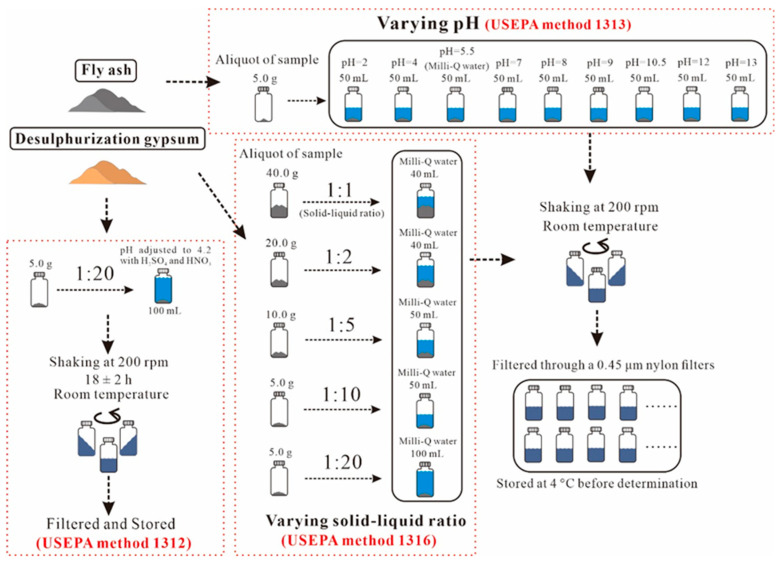
Leaching experiment process following USEPA methods 1312, 1313, and 1316.

The leaching experiments were conducted using recent USEPA methods, including the Leaching Environmental Assessment Framework (LEAF; USEPA methods 1313 and 1316) and the Synthetic Precipitation Leaching Procedure (SPLP; USEPA method 1312). These methods are suitable for modeling industrial waste. A brief thumbnail sketch of the experimental steps is provided in Figure 1, and the detailed steps are shown in the Supplementary Materials.

### 2.3. Field Sample Collection

**Figure 2:**
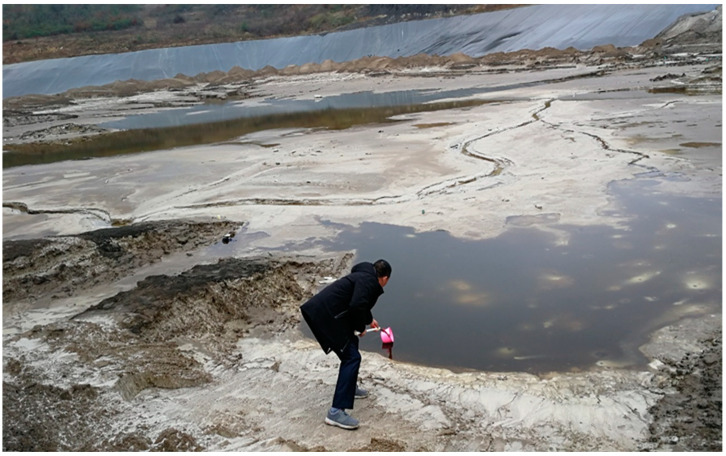
Picture of the sampling of naturally accumulated water from field FGD gypsum dumps (water sample #2).

An FGD gypsum dumpsite near CFPP #16 in northwestern Guizhou Province was selected for an investigation of heavy-metal release in the field. Four sets of naturally stored surface water samples were collected at the central and edge regions of the dumpsite, taking into account factors such as the type of solid byproducts of the CFPP that were deposited. Water sample #1 was a freshwater sample obtained near a fresh gypsum pile at low ground level. Water sample #2 was a dark-yellow water sample obtained between old and new gypsum piles at ground level (Figure 2). Water sample #3 was a freshwater sample obtained from a middle platform near a slope. Water sample #4 was a freshwater sample from a puddle at the junction of a gypsum pile and a slag pile at a middle platform. The pH, salinity, conductivity, and total dissolved solids were measured onsite using a portable water quality tester (Bante 900P pH/Conductivity/DO Meter). Trace elements, including Pb, Cd, and Cr, were determined using ICP-MS.

### 2.4. Quality Assurance and Quality Control

Quality assurance and quality control were performed using blanks, duplicate samples, and certified reference materials to verify the heavy-metal measurements, extraction experiment results, and leaching experiment results. All glassware and bottles were cleaned using 20% HNO_3_ overnight and washed with deionized water before sampling. For heavy-metal analysis, certified reference materials of coal FA (NIST SRM 1633c; GBW 08401), soil (GSS-5), and FGD gypsum (NIST 2429) were used to guarantee analytical quality. The difference between the measurements of Pb, Cd, and Cr contents and the recommended values were found to be <10%.

The accuracy of the experiment was also determined by comparing the sum of the concentrations extracted in Steps 1–5 with the total heavy metals obtained from a single extraction. During the extraction experiment, certified reference materials of coal FA (NIST 1633c), soil (GSS-5), and FGD gypsum (NIST 2429) were also analyzed. The contents of total Pb, Cd, and Cr recovered from the sum of the Pb, Cd, and Cr extracted fractions 1–5 based on a single extraction ranged from 85% to 115%.

Precautions were taken during sampling and analysis to reduce possible contamination. All samples were analyzed in triplicate. All chemicals were guaranteed reagents, and all solutions were prepared using Milli-Q water (18.25 Ω).

## 3. Results and Discussion

### 3.1. Concentrations of Heavy Metals in FA and FGD Gypsum

The concentrations of Pb, Cd, and Cr in the FA and FGD gypsum samples are shown in Table 3.

The concentration of Pb in the FA samples ranged from 31 to 80 mg/kg, which is comparable to the background value for soil in Guizhou Province (35.2 mg/kg) [30]. Quispe et al. also found a similar Pb content in FA from a Brazilian powerplant (49 mg/kg) [8]. The contents of Cr in FA ranged from 104 to 211 mg/kg, which is greater than the soil background value of Cr (95.5 mg/kg). The FA content of Cr (112 mg/kg) reported by Quispe et al. was within this ranges [12], while Zhao et al. found a significantly lower Cr range (43–65 mg/kg) in samples obtained from other areas of China [31]. The FA concentrations of Cd ranged from 0.38 to 3.52 mg/kg, which is somewhat higher than the values reported by Quispe et al. (Cd = 0.8 mg/kg) and Zhao et al. (Cd = 0.56–0.70 mg/kg) [12,31]. The screening value for agricultural soil contamination risk set by Chinese standards is 0.3 mg/kg for Cd [32]; some of the values observed in the present study far exceed this standard. In general, the concentrations of Pb and Cr in FA were at normal levels. In contrast, the concentration of Cd was high and poses a certain environmental risk.

In contrast, the contents of Pb, Cd, and Cr in the FGD gypsum samples were all low (0.03–2.37 mg/kg for Pb, 0.01–0.33 mg/kg for Cd, and 11–62 mg/kg for Cr), being far lower than the relevant soil background values and agricultural soil screening values [32]. This is because only very small proportions of these heavy metals enter FGD gypsum. In the operation of CFPPs, semi-volatile heavy metals such as Pb and Cd volatilize completely at the temperature used in pulverized coal boilers (1300 °C). When the temperature drops below the boiling point after passing through a coal economizer and air preheater, almost all semi-volatile heavy metals adhere to the dust and are captured by the air pollution control device, where they become FA. However, while the boiling points of most semi-volatile heavy-metal compounds are exceeded at the temperatures used in circulating fluidized bed boilers (750–950 °C), certain kinds of heavy-metal compounds still remain. Most of the semi-volatile heavy metal compounds eventually enter the FA, while a small proportion enters the bottom ash. Therefore, for nonvolatile heavy-metal elements such as Cr, even at the high temperatures used in pulverized coal boilers, a considerable amount of Cr does not become volatile and ends up in ash. Zhao et al.’s results regarding the heavy-metal contents of FGD gypsum are relatively consistent with the present study, further supporting this conclusion [19].

### 3.2. Heavy-Metal Migration Potential of FA and FGD Gypsum

#### 3.2.1. Fractional Distribution of Heavy Metals in FA

**Figure 3:**
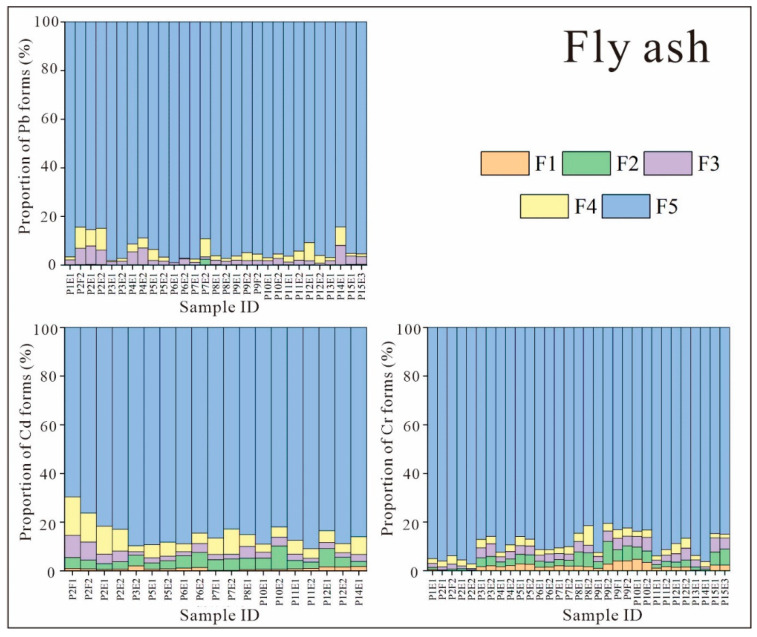
Proportions of heavy-metal forms in the FA samples from CFPPs in Guizhou Province (CFPP ID: P: plant ID; E: fly ash collected from ESP; F: fly ash collected from FF.).

Figure 3 presents the Pb, Cd, and Cr forms found in the FA samples. The main forms of Pb, Cd, and Cr in FA were consistent, with all being present mainly as residues (F5; Figure 3). Heavy metals in this form are very stable and are extremely difficult to release into the natural environment. Although the other four forms accounted for smaller proportions, there were different patterns between the different heavy metals. It should be noted that F1–F4 are known as transportable states, but F2–F4 only partially migrate under very extreme conditions; hence, the proportion of heavy-metal F1 forms is most significant in the real world as they are very active in the environment.

The forms of Pb in FA could be ranked in terms of content as follows: residue state (F5) > reducible state (F4) ≈ oxidizable state (F3) > acid extractable state (F2) ≈ water-soluble state (F1). The percentage of Pb in F5 was 84–99%, while the percentages of Pb in F3 and F4 were low, at 1–8% and 0–9%, respectively. Forms F1 and F2 accounted for very small percentages, with F1 peaking at 0.08% and F2 peaking at 2%. This finding is consistent with previous studies [18,19,31]. In the study of Świetlik et al., no Pb in the F1 and F2 forms was detected in FA, and the proportion of Pb in form F5 ranged from 70% to 86% [18]. In Quispe et al.’s study of a Brazilian powerplant, the proportions of forms F1 and F2 in FA were also close to the detection limit, with Pb in form F5 comprising approximately 77% of the total Pb [19]. Within CFPPs (pulverized coal furnace plants), thermodynamic calculations indicate that PbCl_2_ is the most frequently occurring Pb compound at approximately 900 °C. Above 1000 °C, PbO(g) is the predominant stable Pb compound. At 1200 °C, PbO(g) gradually begins to decompose; then, at the maximum flame chamber temperature (1620 °C), Pb(g) is the most likely form of Pb [33]. Above the maximum temperature, in the cooler parts of the combustion chamber, Pb condenses on FA particles and is converted to PbO. These thermal conditions (melted FA particles) are favorable for the formation of a large number of environmentally persistent states (e.g., residue state) [18]. Unreacted PbO may then form PbSO_4_ (oxidizable state, F3) and PbO_2_ (reducible state, F4) [34]. In contrast, the maximum internal temperature of circulating fluidized bed boiler plants is only about 800 °C; thus, the proportion of Pb in the residual state is significantly lower than that in pulverized coal furnace plants. These results definitively illustrate that several of the powerplant samples with the highest F5 proportions (P2F1, P2E1, P2E2, P4E1, P4E2, and P14E1) were obtained from fluidized bed boiler power plants (Figure 3).

Due to the limitations of the current experimental conditions and experimental methods, it was difficult to determine the low-content forms of Cd in FA. As such, sufficient Cd was unable to be detected in a large proportion of the samples; thus, only samples from some of the powerplants could be analyzed. The proportions of the major forms of Cd in FA were as follows: F5 (83–91%) > F4 (2–11%) ≈ F3 (2–5%) ≈ F2 (2–10%) > F1 (0.1–2.0%). There are large variations between and within previous studies that analyzed Cd forms in FA [19,31]. In Zhao et al.’s study [31], form F3 dominated (~90%), while, in Quispe et al.’s study [19], form F5 was absolutely dominant, although form F5 only accounted for ~50% of the total Cd in some FA samples. Combined with Świetlik et al.’s [18] finding of Cd forms in FA lower than the detection limit, it appears that heavy metals present in low proportions are prone to large measurement deviations due to continuous leaching during extraction experiments.

As for the forms of Cr in FA, F1, F2, F3, and F4 accounted for roughly equal proportions, with F5 being much higher. The Cr forms were ranked as follows: F5 (73–97%) > F4 (2–5%) ≈ F3 (1–7%) ≈ F2 (0.2–10%) ≈ F1 (0–6%). Although, again, F5 was overwhelmingly dominant, it is noteworthy that the F1 form of Cr accounted for the highest percentage of ~6%. Compared to Pb and Cd, the proportion of Cr in form F1 was the highest. Due to the relatively high Cr contents in the FA from some CFPPs in Guizhou Province, the cross-media migration capacity of Cr in FA warrants alarm.

#### 3.2.2. Fractional Distribution of Heavy Metals in FGD Gypsum

**Figure 4:**
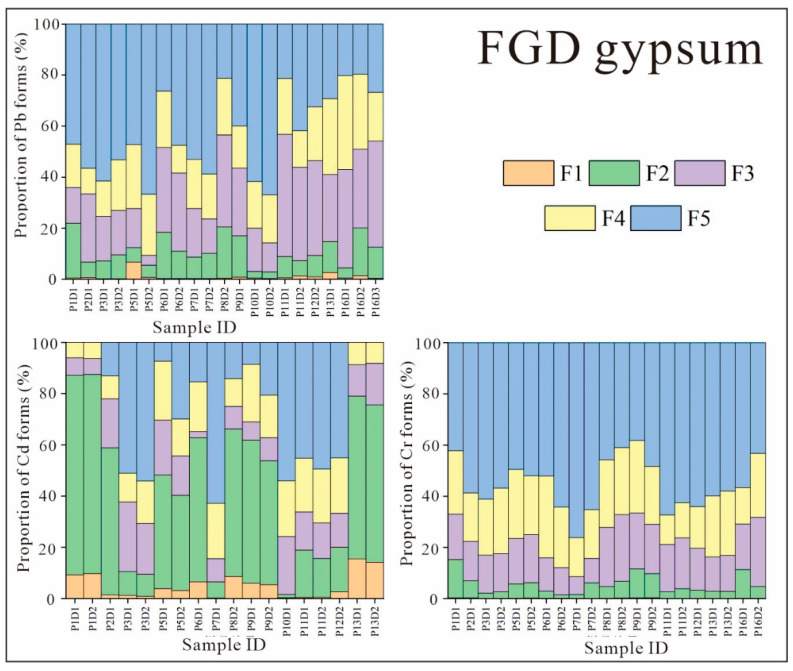
Proportion of different heavy-metal forms in the FGD gypsum samples from CFPPs in Guizhou Province (CFPP ID: P: plant ID; E: fly ash collected from ESP; F: fly ash collected from FF.).

Figure 4 shows the forms of Pb, Cd, and Cr found in the FGD gypsum samples obtained from CFPPs. From the heavy-metal contents reported in Section 3.1, it is clear that only very small proportions of Pb, Cd, and Cr end up in FGD gypsum. These low levels, combined with the issue of stepwise leaching under experimental conditions and the limitations of heavy-metal measurements mentioned previously, resulted in large errors in the recoveries of some of the samples. Hence, some of the forms of heavy metals are not discussed.

In some of the FGD gypsum samples, such as P1D1, P5D1, and P13D1, most of the Cd was present in the mobile phase (F1 + F2 + F3 + F4), which is consistent with the results of Zhang et al. and Hao et al. [35,36]. In other FGD gypsum samples, such as P3D1, P7D1, and P10D1, F5 was the main form of Cr. This greater variation in the forms of Cr in FGD gypsum was also reported by Zhao et al. [20]. This suggests that, in FGD gypsum with a low Cd content, the influences of coal composition, pollution control system operating parameters, and Cd forms in the feedstocks of different CFPPs may lead to very different measurement results. Although the Cd in some FGD gypsum samples is extremely mobile, the very low levels observed in the present study are unlikely to significantly impact the environment.

Although the residual forms of Pb and Cr still accounted for large proportions of the FGD gypsum samples (Figure 4; 61–67% and 38–67%, respectively), these were significantly lower relative to their forms in FA, which is broadly consistent with previous studies [20]. The main reason for this phenomenon is that these less volatile heavy-metal elements, which volatilize into a gaseous state at the high temperatures inside CFPPs, condense and adhere to particulates when the temperature drops. Hence, a large proportion of inactive heavy-metal compounds adhere to particulate matter and are captured by air pollution control devices to form FA. The majority of heavy-metal compounds that escape to wet FGD units are the more active ones, which account for only a small fraction of the total, and they are eventually captured by the desulfurization slurry. Although the release dynamics of such heavy metals in FGD gypsum are relatively high, their levels are low enough to pose little threat to the environment.

### 3.3. Liquid-Phase Transport Capacity of Heavy Metals

#### 3.3.1. Characteristics of Leaching under Different pH Values

**Figure 5:**
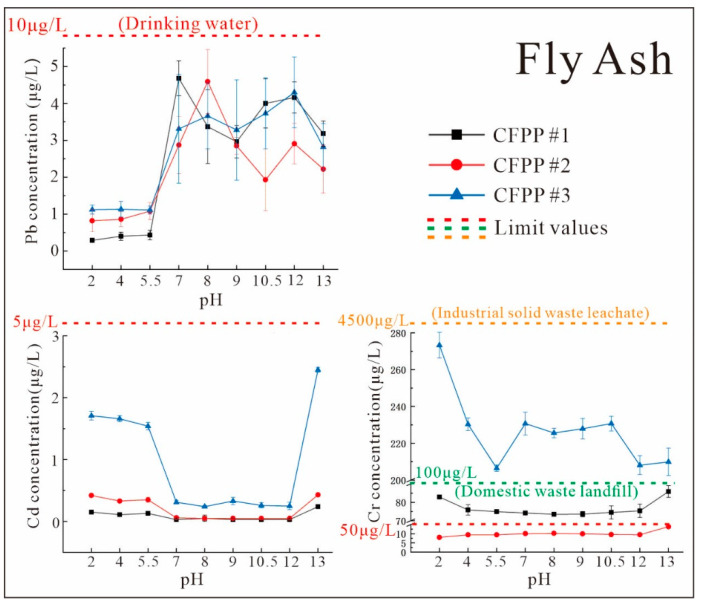
Characteristics of heavy metals leaching from coal FA at various pH values and a constant solid–liquid ratio of 1:10 (The dashed lines indicate the pollution concentration limits for different standards.).

**Figure 6:**
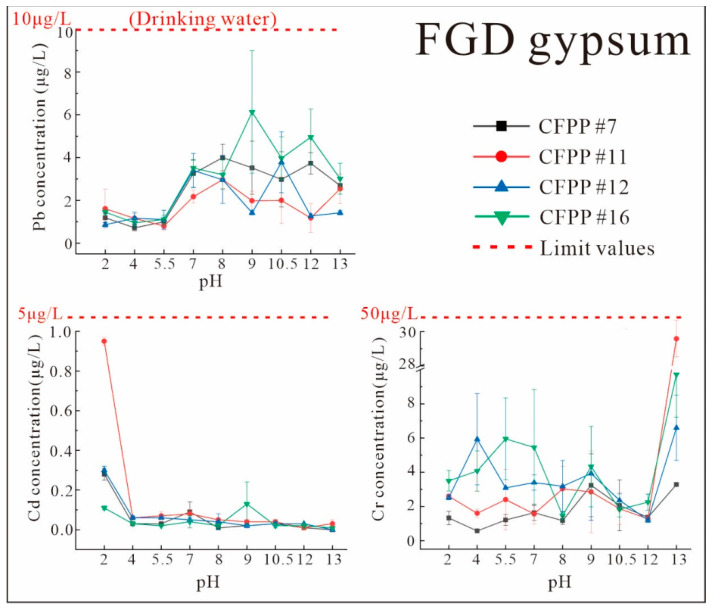
Characteristics of heavy metals leaching from FGD gypsum at various pH values and a constant solid–liquid ratio of 1:10 (The dashed lines indicate the pollution concentration limits for the different standards.).

Figure 5 and Figure 6 show the variations in the levels of Pb, Cd, and Cr in FA and FGD gypsum leachate according to the extracting solution pH, with the relevant national water quality or wastewater discharge limits presented in each graph. The Chinese standard limits for heavy metals in drinking water are used as a reference [37].

Figure 5 and Figure 6 show the variations in the Pb content of extracts from FA and FGD gypsum under different pH conditions. Although there were large differences in the Pb contents and forms of the FA and FGD gypsum (Table 3; Figure 3), the Pb concentrations in the extracts were generally comparable and showed similar patterns of variation with pH (Figure 5 and Figure 6).

Overall, the Pb levels in the extracts were low (0–2 μg/L) at low pH values (2–5.5, acidic environment). They showed a clear increasing trend at pH = 5.5–7 (1.5–4 μg/L) and remained largely stable (2–6 μg/L) at pH = 7–13. This is mainly due to the fact that Pb is easily enriched in solids and its mobility is related to the solubility of its associated minerals, which are mainly water-soluble Pb(OH)_2_, $\mathrm{Pb}{\left(\mathrm{OH}\right)}_{3}^{-}$, and PbOH^+^ under alkaline conditions. $\mathrm{Pb}{\left(\mathrm{OH}\right)}_{3}^{-}$ also increased significantly with alkalinity [38], resulting in higher Pb concentrations under alkaline conditions. According to the Pollution Control Standard for Domestic Waste Landfills [39], the allowable Pb concentration limit in the leachate of industrial solid waste destined for landfill after treatment was 250 μg/L. In the present study, the Pb contents in all pH ranges were well below this value (Figure 5 and Figure 6) and were even below the allowable Pb concentration for domestic drinking water (10 μg/L) in China. This indicates that cross-media migration of Pb from naturally stockpiled FA and FGD gypsum to the liquid phase is extremely unlikely, even under extreme environmental conditions.

The low initial Cd contents in FA and FGD gypsum resulted in lower Cd concentrations in the dewatered leachate with no clear pattern of variation. In Figure 5 and Figure 6, the Cd concentrations in FA leachate were higher in acidic (pH = 2–5.5) and very strongly alkaline environments (pH = 13) than in neutral and weakly alkaline environments (pH = 7–12). On the other hand, in FGD gypsum leachate, high concentrations of Cd were only observed under very acidic conditions (pH = 2), with little difference under other pH conditions. However, due to the detection limits, these variations may not be significant. The peak Cd contents under strongly acidic and strongly alkaline conditions were determined by the dissolution of Cd compounds under acidic conditions and the formation of anionic hydroxyl complexes under strongly alkaline conditions. Similarly, the Cd concentrations in all leachates were below the limits for industrial waste leachate (150 μg/L) and domestic drinking water (5 μg/L). Therefore, Cd is not easily released into the environment.

As seen in Figure 5 and Figure 6, FA and FGD gypsum showed various degrees of Cr release. The Cr concentrations in the FA drippings from the three CFPPs showed large differences. In the samples from CFPP #3, Cr levels in the drippings in all pH ranges (206–273 μg/L) were greater than those from CFPP #1 (74–86 μg/L) and CFPP #2 (8–14 μg/L). This is due to the much higher proportion of removable dynamic Cr in CFPP #3 compared with CFPP #1 and CFPP #2, which allowed for more Cr to be released in the drippings release. The Cr levels in all three plants were well below the limit for industrial solid waste treatment leachate (total Cr < 4500 μg/L); however, unlike the other heavy metals, the Cr levels in the leachates from CFPP #1 and CFPP #3 exceeded the limit for drinking water (50 μg/L), while those from CFPP #1 even exceeded the standard for domestic waste landfill [39]. This indicates that Cr contamination of FA produced at CFPP #1 may occur when the plant is centrally landfilled, which requires strict control and screening. These findings also indicate that pH variation is not a key factor in determining the release of Cr from FA; this is mainly determined by the fugitive form of Cr.

The Cr content of the FGD gypsum leachate only showed a sharp increase under strongly alkaline conditions (pH = 13), with CFPP #11 showing a 10-fold increase. Although the Cr concentration limits for drinking water were not exceeded at any of the pH values, such a large spike at pH = 13 suggests that, under very strong alkaline conditions, coal combustion byproducts may contribute to Cr release (Figure 6).

Environmental monitoring data for 2018–2021 show that the annual average pH of precipitation in different areas in Guizhou Province ranged from 6.24 to 7.89, with a very low frequency of acid rain [40,41,42,43]. As can be seen in Figure 5 and Figure 6, heavy metals are extensively leached out in this pH range. Thus, although the total leaching amount is small (except for Cr), it should be a cause for alarm.

#### 3.3.2. Characteristics of Leaching under Different Solid–Liquid Ratios

**Figure 7:**
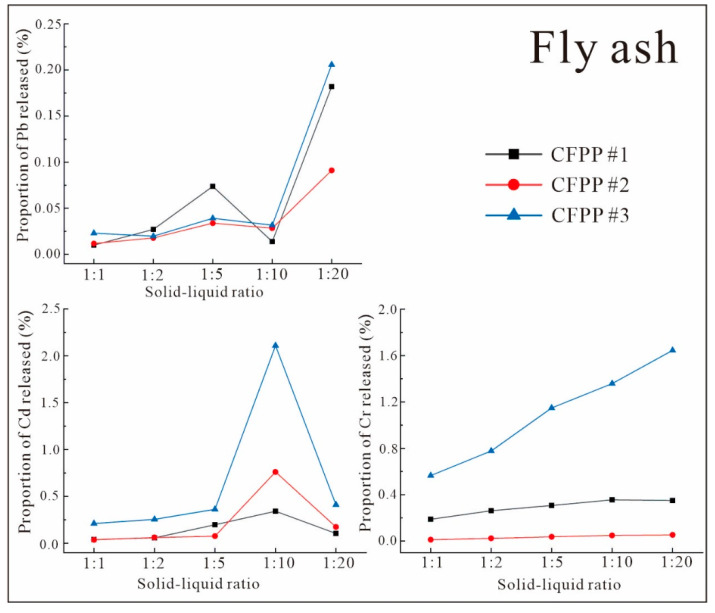
Heavy-metal release fractions from coal FA with various solid-to-liquid ratios.

**Figure 8:**
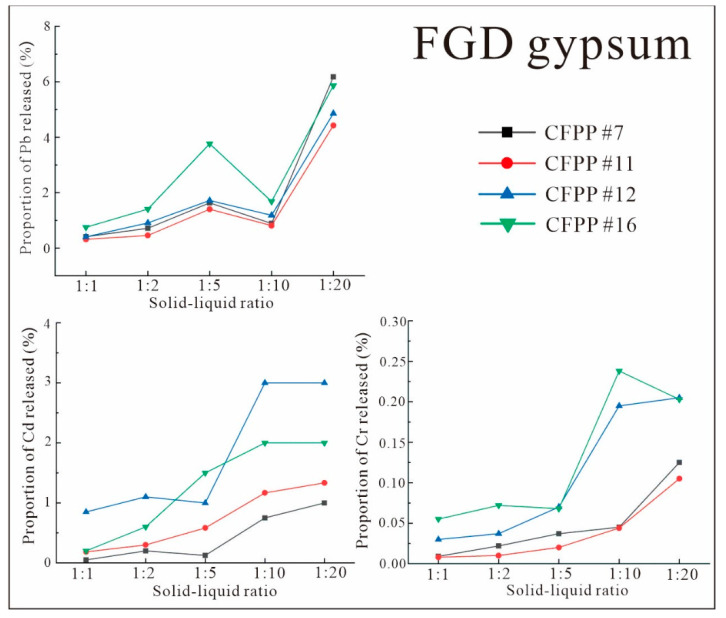
Heavy-metal release fractions from FGD gypsum with various solid-to-liquid ratios.

The release fractions of Pb, Cd, and Cr from FA and FGD gypsum are shown in Figure 7 and Figure 8, respectively, for different solid-to-liquid ratios.

From Figure 7 and Figure 8, it can be seen that the release patterns of each heavy metal from FA and FGD gypsum at various solid-to-liquid ratios were generally consistent, whereby the release rate increased as the solid-to-liquid ratio decreased. Pb, Cd, and Cr showed high release rates at solid-to-liquid ratios of 1:10 and 1:20. According to previous studies [44], this pattern is similar to Hg. This indicates that the dominant effect of the solid-to-liquid ratio on heavy-metal release from CFPP byproducts is based on solubility. Specifically, the release rate of solid heavy metals is low at higher solid-to-liquid ratios (i.e., less leachate) due to the solubility limits of heavy metals. In contrast, as the solid-to-liquid ratio decreases, the proportion of leaching reagent increases and the fraction of solids that can dissolve heavy metals increases, leading to a continuous increase in the release rate. This should remain relatively stable when the dissolution limit is reached and will no longer increase with increases in the solid-to-liquid ratio. However, the particular solid-to-liquid ratio that maintains a stable release rate is determined by factors such as the heavy-metal binding forms and concentration of the solid components, i.e., different FAs may be stable under different solid-to-liquid ratio conditions. For example, the peak of the release rate of Cd at a solid-to-liquid ratio of 1:10 in Figure 7 is due to the limitations of total Cd and the nature of FA Cd. In addition, in Figure 5 and Figure 7, the release rates of each FA and FGD gypsum sample varied considerably between each heavy metal. For example, the highest Pb release rate from FGD gypsum was close to 6% (Figure 7), while that of Cd from FA was <0.007% (Figure 5). This is because the proportions of heavy metals in migratable states differed greatly between samples, resulting in significant differences in the release rates. Therefore, the variation in the solid-to-liquid ratio did not result in additional heavy-metal release, and the upper limit of heavy-metal release was determined by the nature of the sample itself.

### 3.4. Heavy-Metal Release under Indoor Simulated Rainfall and Field Sampling

To enable a visual comparison of heavy metals leaching from FA and FGD gypsum under natural conditions (USEPA Method 1312), we determined heavy-metal contents under a laboratory simulated leaching experiment and field leachate sampling. The results are shown in Table 4 and Table 5. 

Consistent with the results of a previous study, it can be seen in Table 4 that the Cr in some FA samples exceeded the emission limits for heavy metals due to the proportion of Cr in a transportable state and the nature of the heavy metal. For example, the Cr content in the leachate of FA from CFPP #3 after simulated rainfall far exceeded the Chinese drinking water quality limit (50 μg/L) and was close to the landfill water pollutant discharge limit (100 μg/L). However, aside from this, all hazardous heavy metals in FA and FGD gypsum in this study did not pose leaching release risks under simulated rainfall.

A comprehensive analysis of water quality parameters from water samples collected in the field (Table 5) indicated that the water quality could be ranked from worst to best as follows: #2, #1, and then #3 and #4 (which were similar). From the reference data in Table 5, it can be seen that, although the water quality parameters (total dissolved solids, conductivity) of the four groups of water samples were far from meeting the relevant standards for domestic drinking water, in general, the contents of most heavy metals met the requirements (except for Cr in water sample #2). The longer leaching time of sample #2, which was collected at the junction of old and new gypsum yard in the lower terrain, was long enough for sufficient leaching of FGD gypsum. Hence, the values are more representative of long-term heavy-metal leaching from piles and concentrated landfill coal combustion byproducts subjected to natural rainfall.

In addition, although it is clear that water sample #2 had much poorer water quality parameters than the other three samples, not all of the heavy metals in the sample were significantly higher than the other water samples. For example, the Pb content in #2 was lower than that in #1. This indicates that the liquid-phase transfer of some heavy metals quickly reached an upper limit that is much lower than the drinking water limit (Table 5). In contrast, the Cr content (64 μg/L) exceeded the limit (50 μg/L), which is consistent with the results of the leaching experiment with simulated rainfall (Table 4). This indicates that the liquid-phase transport capacity of Cr in natural stockpiles and concentrated landfills of coal combustion byproducts poses somewhat of an environmental threat.

## 4. Conclusions

This study investigated the leaching characteristics and environmental release potential of Pb, Cd, and Cr from the solid byproducts of CFPPs under natural stockpiling conditions. FA and FGD gypsum samples were obtained from 16 CFPPs in Guizhou Province, and a combination of field analyses and indoor experiments were performed. The main conclusions are as follows:

1. Pb, Cd, and Cr were all predominantly in the residual state in FA, indicating that there is little release of these elements from FA. The forms of Pb, Cd, and Cr in FGD gypsum did not show a clear pattern, and their total contents were extremely low, posing low threats to the environment.
2. The release rates of Pb, Cd, and Cr from FA and FGD gypsum into leachate varied according to pH. The release rates of Pb and Cd from FA and from FGD gypsum were very low in all experiments. However, the release of Cr from FA and FGD may have been greater due to the high proportion of water-soluble states observed in some samples; this was confirmed by both indoor simulations and field observations.

## Figures and Tables

**Table 1:** Detailed information about the solid samples collected from CFPPs in Guizhou Province, China.

CFPP ID	Location in Guizhou	Boiler Horsepower	Boiler Type ^1^	Pollutant Control Facilities ^2^	Sample Type	Sample ID ^3^
#1	Central	3 × 200 MW	PC	SCR + ESP + WFGD	FA from ESP	P1E1, P1E2
					FGD gypsum	P1D1, P1D2
#2	West	1 × 300 MW	CFB	SNCR + ESP-FF + WFGD	FA from ESP and FF	P2E1, P2E2, P2F1, P2F2
					FGD gypsum	P2D1
#3	Central	4 × 300 MW	PC	SCR + ESP + WFGD	FA from ESP	P3E1, P3E2
					FGD gypsum	P3D1, P3D2
#4	Northwest	2 × 150 MW	CFB	ESP	FA from ESP	P4E1, P4E2
#5	Northwest	4 × 300 MW	PC	SCR + ESP + WFGD	FA from ESP	P5E1, P5E2
					FGD gypsum	P5D1, P5D2
#6	East	2 × 300 MW	PC	SCR + ESP + WFGD	FA from ESP	P6E1, P6E2
					FGD gypsum	P6D1, P6D2
#7	North	2 × 660 MW	PC	SCR + ESP + WFGD	FA from ESP	P7E1, P7E2
					FGD gypsum	P7D1, P7D2
#8	West	2 × 600 MW	PC	SCR + ESP + WFGD	FA from ESP	P8E1, P8E2
					FGD gypsum	P8D1, P8D2
#9	West	2 × 660 MW	PC	SCR + ESP-FF + WFGD	FA from ESP and FF	P9E1, P9E2, P9F1, P9F2
					FGD gypsum	P9D1, P9D2
#10	Northwest	4 × 300 MW	PC	SCR + ESP-FF + WFGD	FA from ESP	P10E1, P10E2
					FGD gypsum	P10D1, P10D2
#11	Northwest	8 × 300 MW	PC	SCR + ESP-FF + WFGD	FA from ESP	P11E1, P11E2
					FGD gypsum	P11D1, P11D2
#12	Central	2 × 600 MW	PC	SCR + ESP + WFGD	FA from ESP	P12E1, P12E2
					FGD gypsum	P12D1, P12D2
#13	West	4 × 600 MW	PC	SCR + ESP + WFGD	FA from ESP	P13E1, P13E2
					FGD gypsum	P13D1, P13D2
#14	Central	2 × 150 MW	CFB	ESP	FA from ESP	P14E1
#15	North	4 × 300 MW	PC	SCR + ESP-FF + WFGD	FA from ESP	P15E1, P15E2, P15E3
#16	Northwest	4 × 300 MW	PC	SCR + ESP-FF + WFGD	FGD gypsum	P16D1, P16D2, P16D3, P16D4

^1^ PC: pulverized coal-fired boilers, CFB: circulating fluidized bed boilers; ^2^ SCR: selective catalytic reduction; SNCR: selective noncatalytic reduction; ESP: electrostatic precipitator, FF: fabric filter; WFGD: wet flue gas desulfurization; ^3^ P: CFPP ID; E: fly ash collected from ESP; F: fly ash collected from FF.

**Table 2:** BCR sequential extraction method.

Step	Extraction Reagent	Experimental Conditions	Description
1	Deionized water	Room temperature, 16 h	Water soluble
2	0.5 M CH_3_COOH	Room temperature, 16 h	Acid soluble
3	0.5 M HONH_2_HCl	Room temperature, 16 h	Reducible
4	H_2_O_2_; 1 M NH_4_C_2_H_3_O_2_	85 °C; room temperature, 20 h	Oxidizable
5	HCl, HNO_3_, HF, HClO_4_	195 °C	Residual

**Table 3:** The concentrations of heavy metals in FA and FGD gypsum samples (unit: mg/kg).

CFPP ID	Pb		Cd		Cr	
	FA	FGD Gypsum	FA	FGD Gypsum	FA	FGD Gypsum
#1	31	0.55	0.38	0.33	211	33
#2	37 (38) ^1^	0.50	0.47 (0.46) ^1^	0.31	199 (193) ^1^	40
#3	35	0.03	0.73	0.03	152	50
#4	55	/ ^2^	1.00	/ ^2^	104	/ ^2^
#5	64	0.43	1.12	0.11	162	62
#6	37	2.37	0.87	0.05	180	36
#7	57	1.13	3.52	0.04	186	27
#8	52	1.25	0.49	0.16	108	42
#9	58 (80) ^1^	0.66	0.65 (1.07) ^1^	0.22	131 (146) ^1^	28
#10	73	1.52	1.99	0.03	125	11
#11	37	0.99	0.60	0.06	164	55
#12	41	0.93	0.79	0.02	170	35
#13	32	0.31	0.44	0.27	156	35
#14	44	/ ^2^	2.28	/ ^2^	136	/ ^2^
#15	53	/ ^2^	2.33	/ ^2^	163	/ ^2^
#16	/ ^2^	0.66	/ ^2^	0.01	/ ^2^	25

^1^ Data in brackets are the heavy-metal contents of FA collected from the fabric filter, while data outside of brackets are the heavy-metal contents of FA collected from the electrostatic precipitator; ^2^ /: no data.

**Table 4:** Heavy-metal release from FA and FGD gypsum under simulated rainfall leaching conditions.

CFPP ID	Sample Type	Concentrations of Heavy Metals (μg/L)		
		Pb	Cd	Cr
CFPP #1	Fly ash	2.731	0.029	33.778
CFPP #2	Fly ash	2.207	0.030	4.836
CFPP #3	Fly ash	3.365	0.136	97.123
CFPP #7	Desulfurization gypsum	3.325	0.027	1.856
CFPP #11	Desulfurization gypsum	2.006	0.034	1.588
CFPP #12	Desulfurization gypsum	2.304	0.049	1.334
CFPP #16	Desulfurization gypsum	3.238	0.010	2.047
Drinking water limits in China (μg/L) [39]		10	5	50
Drinking water limits in the US (μg/L) [45]		15	5	100
Emission limits for water pollutants from landfills in China (μg/L)		100	10	100

**Table 5:** Water quality parameters and heavy metals contents of water samples collected close to combustion byproduct landfill.

Water Quality Parameters	Water Sample #1	Water Sample #2	Water Sample #3	Water Sample #4	Drinking Water Limit in China
pH	7.47	7.48	8.85	9.39	6.5~8.5
Salinity (psu)	4.35	34.26	1.26	1.24	/
Electrical conductivity (mS)	8.21	55.8	2.57	2.55	2
Total dissolved solids (mg/L)	3990	14,000	1254	1238	1000
Pb content (μg/L)	4.25	3.91	0.41	0.35	10
Cd content (μg/L)	0.57	1.98	1.30	1.31	5
Cr content (μg/L)	19.72	64.07	42.39	41.78	50

## Data Availability

Not applicable.

## Supplementary Materials

The following supporting information can be downloaded at: https://www.mdpi.com/article/10.3390/ijerph191912617/s1, containing supporting methods of sequential extraction experiment details and heavy-metal leaching experiment details.
