# Supplementary material for: Concentrations, Speciation, and Potential Release of Hazardous Heavy Metals from the Solid Combustion Residues of Coal-Fired Power Plants
Source: Int J Environ Res Public Health. 2022 Oct 2;19(19):12617. doi: 10.3390/ijerph191912617 (PMC9566641; doi:10.3390/ijerph191912617)
Supplement: Supplementary file 1 [file ijerph-19-12617-s001.zip › ijerph-1912366-supplementary.pdf]

Supporting Information

Concentrations, Speciation, and Potential Release of Hazardous Heavy Metals from Solid Combustion Residues of Coal-fired Power Plants

Yiming Huang [1,2,3], Jinling Liu [2], Guan Wang [1,2], Xiangyang Bi [2], Guangyi Sun [4], Xian Wu [4], Qingfeng Wang [1,*] and Zhonggen Li [1,*]

[1] School of Resources and Environment, Zunyi Normal College, Zunyi 563006, China

[2] School of Earth Sciences, China University of Geosciences, Wuhan 430074, China

[3] MOE Laboratory for Earth Surface Processes, College of Urban and Environmental Sciences, Peking University, Beijing 100871, China

[4] State Key Laboratory of Environmental Geochemistry, Institute of Geochemistry, Chinese Academy of Sciences, Guiyang 550081, China

*Correspondence Author

Email: qingfeng_424@163.com(Wang Q.); lizhonggencn@126.com (Li Z.)

Tel: +86 136 0851 7028

SUPPORTING METHODS.

Sequential Extraction Experiment Details

The details information of sequential extraction method:

Step 1: Weigh 1 g of solid sample into a centrifuge tube and add 20 mL of deionised water, then shake and extract at room temperature for 16 h. Centrifuge and filter (0.45 μm membrane) the sample to remove the supernatant into a polyethylene bottle, acidify and store at 4 °C for testing.

Step 2: Add 40 mL of 0.5 M acetic acid solution to the remainder of the centrifuge tube obtained in Step 1, shake, centrifuge and filter the supernatant, acidify and store.

Step 3: Add 40 mL of 0.5 M hydroxylamine hydrochloride to the remainder of the centrifuge tube obtained in Step 2, shake, centrifuge, filter and store the supernatant.

Step 4: Slowly add 10 mL of hydrogen peroxide to the remaining species in the centrifuge tube and digest at room temperature for 1 h with constant shaking. Continue the digestion at 85 °C for 1 h with constant shaking for the first 0.5 h. Open the lid and heat the water bath until the volume is reduced to < 3 mL, then add another 10 mL of hydrogen peroxide solution and continue digestion at 85 °C for 1 h with constant shaking for the first 0.5 h. Open the lid and heat the water bath until the volume is reduced to 1 mL. Add 50 mL of 1 M ammonium acetate solution to the cold wet residue, shake, centrifuge, filter to remove the supernatant, and acidify for storage.

Step 5: Evaporated the residue of the centrifuge tube in a water bath, thoroughly transfer it to a vessel, grind and weigh 50 mg of it into a PTFE crucible, wet with water, then add 3 mL, 2 mL, 1 mL, and 5 mL of hydrochloric acid (HCl), nitric acid

(HNO3), perchloric acid (HClO4) and hydrofluoric acid (HF), respectively, and place on an electric hot plate and heat until the white smoke of HClO4 is exhausted. Add acid depending on the digestion and repeat the above steps. After digestion, add 1mL of HCl, heat until the salts are dissolved, remove and cool, add water up to a volume of 10 mL, and store for testing. Measure the Pb, Cd and Cr in all solutions by ICP-MS.

Heavy Metal Leaching Experiment Details

The details information of leaching experiment method:

USEPA methods 1313 and 1316 were used to investigate the leaching characteristics of FA and FGD gypsum at different pHs and liquid/solid ratios. USEPA method 1313 adjusts the pH of the leachate to nine gradients (2, 4, 5.5, 7, 8, 9, 10.5, 12, and 13) with HNO3 and KOH and takes a 20 g sample with a liquid-to-solid ratio of 10:1 (w/v, g/mL). The samples were shaken for 24 h, centrifuged, and filtered to determine the supernatant. The USEPA 1316 method was studied using five different liquid-to-solid ratios (0.5, 1.0, 2.0, 5.0, 10 w/v), with samples added to deionized water and shaken for 24 h, then filtered and analysed for determination.

The USEPA Method 1312 method simulates the release of FA and FGD gypsum under rainfall conditions. The pH of the leachate was adjusted to 4.2 with H2SO4 and HNO3, shaken for 18 ± 2 h at a liquid-to-solid ratio of 20:1, and filtered for determination. For all three experiments, three experimental blanks (without solid samples) were made for comparison with the experimental blanks and were deducted from the sample results.

Trace elements in the above water samples were determined by ICP-MS.
